# The role of internet addiction and academic resilience in predicting the mental health of high school students in Tehran

**DOI:** 10.1186/s12888-024-05853-6

**Published:** 2024-06-04

**Authors:** Maryam Latifian, Mahta Alsadat Aarabi, Sahar Esmaeili, Kianoush Abdi, Ghoncheh Raheb

**Affiliations:** 1 Substance Abuse and Dependence Research Center, University of Social Welfare and Rehabilitation Sciences, Tehran, Iran; 2 Department of Counselling, University of Social Welfare and Rehabilitation Sciences, Tehran, Iran; 3 Faculty of Social Work, University of Calgary, Calgary, Canada; 4 Department of Rehabilitation Management, University of Social Welfare and Rehabilitation Sciences, Tehran, Iran

**Keywords:** Internet addiction, Academic resilience, Mental health, Students, Tehran

## Abstract

**Background:**

The World Health Organization defines mental health as a combination of two dimensions: the negative dimension, or negative mental health, which indicates the presence of mental disorders, symptoms, and problems, and the positive dimension, or positive mental health, which includes emotions and positive personal characteristics such as self-esteem, resilience against environmental challenges, a sense of integrity, and self-efficacy. The aim of the present study was to investigate the role of internet addiction and academic resilience in predicting the mental health of high school students in Tehran, Iran.

**Method:**

The research method employed was a survey. 758 people participated in the study, and the samples consisted of high school students in Tehran during the academic year 2022–2023. The process of collecting information was carried out by distributing the questionnaire link through virtual networks and schools. The research utilized Young’s Internet Addiction Test, Samuels’ Academic Resilience Inventory, and Goldberg’s Mental Health Questionnaire as the research tools. Statistical tests, including Pearson’s correlation and multiple regression analysis, were employed to investigate the relationships between variables.

**Result:**

The effect of internet addiction on mental health (ß=0.39) is negative and significant at the 0.001 level, while the effect of academic resilience on mental health (ß=0.66) is positive and significant at the 0.001 level. These two variables collectively predict 53% of the variance in students’ mental health. This indicates that as internet addiction increases among students, their mental health significantly decreases, whereas higher levels of academic resilience correspond to higher mental health.

**Conclusions:**

This study has elucidated the role of internet addiction and academic resilience in predicting the mental health of high school students in Tehran. Given the significance of adolescent mental health, it is imperative for healthcare professionals and other stakeholders to develop intervention and prevention models to address mental health crises and plan for the enhancement of adolescent mental health.

## Introduction

Mental health is considered one of the determinants of people’s general health. The concept encompasses feelings of well-being, self-efficacy, autonomy, resilience, a sense of belonging, self-actualization, and the realization of intellectual and emotional potential [1, 2]. The World Health Organization defines mental health as an integration of two dimensions, negative and positive. The negative dimension, or negative mental health, indicates the presence of mental disorders, symptoms, and problems, while the positive dimension, or positive mental health, encompasses emotions and positive personal characteristics such as self-esteem, resilience against environmental challenges, a sense of integrity, and self-efficacy [3]. Treating mental illnesses and improving mental health is one of the most important goals of the World Health Organization (WHO) [4, 5].

Adolescence is a period of life associated with the onset or exacerbation of psychological problems and disorders [6, 7]. According to reports from the World Health Organization, approximately 10–20% of adolescents meet the necessary criteria for diagnosing mental disorders [8, 9]. Unfortunately, in many cases, these problems persist without diagnosis [10–12]. If psychological problems are not diagnosed and treated promptly, they can persist chronically into later stages of life and adversely affect a person’s quality of personal and social life. Additionally, they can hinder the attainment of expected capabilities, such as personal growth and education [13, 14]. Adolescence is one of the most critical periods in a person’s life and is associated with biological, psychological, and social changes. For this reason, adolescents may experience various crises that can lead to behavioral problems [15, 16]. Adolescence, as a critical phase in life, confronts adolescents with extensive changes, including physical changes, reevaluation of beliefs and values, and alterations in the quality and scope of social relationships. On the other hand, these inevitable changes also increase the likelihood of engaging in risk behaviors. Therefore, the importance of paying attention to high-risk behaviors during adolescence cannot be overstated [17].

The Internet has become an essential part of our daily lives, particularly for teenagers and young adults. However, there is also a growing concern about determining the threshold for excessive Internet use and identifying when it transitions into addiction [18]. Growing up in a digital world entails risks, especially for the youngest members of society [19]. One of the common high-risk behaviors is Internet addiction [20]. Adolescence, due to its vulnerability to addiction, is a critical period during which the pattern of excessive Internet use is more likely to proliferate compared to adults [21]. With the widespread popularity of smartphones across the globe, access to the Internet has also significantly increased. The number of Internet users worldwide has reached 3.9 billion people, and the rate of Internet usage in developing countries has surged from 7.7 to 43.4% between 2005 and 2018 [22]. For instance, the prevalence of Internet addiction among high school students in Taiwan was reported to be 24.4% in 2020 [23]. Iran is not an exception to this trend, as statistics indicate a significant increase in the number of Internet users in recent years, with a 25-fold increase reported [24]. Furthermore, another study found that the overall prevalence of Internet addiction among Iranian students was 31.51% [25]. Over the past two decades, children and teenagers have exhibited a high prevalence of Internet addiction [26]. Gomez et al.‘s study on the problematic use of the Internet among Spanish teenagers reveals that a significant proportion of teenagers have experienced deviant behaviors as a result of excessive Internet and social media usage. Consequently, there is a pressing need to empower parents to moderate their children’s Internet usage [27].

Some individuals exhibit remarkable flexibility in coping with stressful factors, while others struggle, placing their mental health at risk. A review of studies has highlighted resilience as a key factor influencing individuals’ mental health. The concept of resilience gained prominence in the work of Werner in the 1970s, who defined it as the ability of individuals to maintain biological and psychological equilibrium in adverse conditions [28]. In other words, resilience refers to the positive capacities and characteristics that enable individuals to confront environmental challenges positively and shield them from stress-related mental disorders [29].

The characteristics of resilient people include flexibility, emotional intelligence, emotional insight, tenacity, hope, positive outlook, and self-confidence. These traits contribute to the development of coping skills and help protect and support individuals against various problems [30]. One of the dimensions of resilience in the educational environment is academic resilience. Students who possess academic resilience, despite facing social, cultural, and economic challenges, are able to achieve high levels of success in education [31]. Academic resilience refers to high levels of motivation for progress and performance, despite the events and stressful conditions that students face at school [5]. Bagheri et al. conducted a study to examine the influence of Internet addiction, mindfulness, and resilience on the mental health of students during the 2019 coronavirus pandemic. Their findings revealed that Internet addiction during the COVID-19 outbreak negatively impacted students’ mental health, increasing their susceptibility to depression and anxiety. The utilization of the Internet, particularly during adolescence, can significantly influence an individual’s emotional and psychological well-being [32]. This study was conducted during the COVID-19 epidemic, a period characterized by students’ reliance on virtual education and absence from physical school settings. The study aimed to examine the impact of Internet addiction, mindfulness, and resilience on the mental health of students within the context of the COVID-19 pandemic.

Mousavi et al.‘s study, titled ‘The Prevalence of Addiction to Social Networks and its Relationship with Depression, Anxiety, and Stress among Iranian Users,’ revealed a high prevalence of social network addiction among the Iranian population and its significant correlation with depression, anxiety, and stress [33]. Additionally, findings from a systematic review study conducted by Mesman et al. indicated that higher levels of resilience are associated with fewer mental health issues [34].

Based on the aforementioned studies, it is evident that the high prevalence of internet addiction worldwide, particularly among adolescents—a critical stage in life—necessitates an examination of its role in predicting their mental health. Furthermore, academic resilience emerges as a crucial issue among high school students, and its impact on predicting their mental well-being has been explored. However, given the lack of knowledge and research in the field of adolescent mental health, coupled with the paramount importance of the studied phenomenon and its dependency on sociocultural factors and living environments, there is a pressing need for attention to be directed towards this issue. This is particularly pertinent in the context of Tehran, where such studies are lacking, highlighting the urgency of addressing this gap. The examination of the role of internet addiction and academic resilience in predicting the mental health of high school students in Tehran is expected to provide essential evidence for mental health caregivers and managers involved in mental health interventions. By understanding the factors influencing mental health in this population, targeted services can be developed to improve their living conditions. Ultimately, this research aims to enhance the quality of life for these adolescents. Therefore, this research was conducted to investigate the role of internet addiction and academic resilience in predicting the mental health of high school students in Tehran, Iran.

## Methods

### Study design

This study was conducted using a quantitative survey method (descriptive-analytical).

### Participants and sample size

The participants of this research were all students of the high school in Tehran, Iran, the year 2022–2023, of which 758 students were available by sampling method and by placing the link of the questionnaire in groups of social media networks of schools completed the questionnaires and thus participated in the study. The inclusion criteria were: (1) being a student in the high school (2), having access to the Internet and virtual space (3), living in Tehran (4), age range from 14 to 18 years (5), not having experience of substance use (6), not having a chronic medical disease (7), not having a history of psychiatric disease and (8) willingness to participate in the research. The most important exclusion criteria was that the participant lost his/her desire to cooperate in the research.

### Instruments

In this research, in addition to the demographic information checklist, Young’s internet addiction test, Samuels’ academic resilience inventory and Goldberg’s mental health questionnaire were used.

#### Young’s internet addiction test

Kimberly Young’s Internet Addiction test was used to measure Internet addiction. This questionnaire was created by Kimberly Young in 1998 and is one of the most reliable questionnaires in the field of Internet addiction. This questionnaire is designed in the form of 20 items and scored by the Likert method. This tool measures different aspects of Internet addiction and determines whether excessive use of the Internet affects different aspects of a person’s life or not. The range of scores for this test is from 20 to 100, and a score higher than 40 indicates dependence on the Internet [35]. The scores obtained for each person were classified into three groups: (A) Normal internet users, (B) Users experiencing problems due to excessive use, and (C) Addicted users whose excessive use has led to dependency and requires treatment. Primary examinations into the validity of the IAT have shown strong internal consistency (α = 0.90–0.93) and good test-retest reliability (*r* = 0.85) values [7–12, 36]. In Iran, Mohagheghi et al. investigated the psychometric properties of the IAT tool, and the results of their study showed that the reliability of the questionnaire was acceptable for 20 questions, with a Cronbach’s alpha coefficient of 0.93. The face and content validity were determined using the Delphi method and by incorporating the opinions of specialists in the field of internet use [37]. In the present study, Cronbach’s alpha for this tool was found to be 0.84.

#### Samuels academic resilience inventory

The Samuels and Woo (2009) questionnaire was utilized to measure academic resilience [38]. This questionnaire, developed by Samuels in 2004 and later expanded upon in a study published in 2009, assesses academic resilience across three dimensions: communication skills, future orientation, and problem-oriented/ positive orientation. The original version of this questionnaire consists of 40 questions. However, after standardization for use in Iran, the number of questions was reduced to 29. The questionnaire employs a 5-point Likert scale, ranging from ‘completely disagree’ to ‘totally agree.’ A higher score on this scale indicates greater academic resilience. In his research, Samuels reported the reliability and validity of this questionnaire as 76%. Furthermore, Soltaninejad et al. standardized the questionnaire for use in Iran [39, 40]. In Soltaninejad et al.‘s study, the psychometric properties of the Samuels Academic Resilience inventory were investigated to evaluate its suitability for Iranian students. The study involved two samples of students. The results indicated that this tool demonstrates acceptable internal consistency, with Cronbach’s alpha values for the factors ranging between 0.63 and 0.77 in the student samples [41]. In the present study, Cronbach’s alpha for this tool was found to be 0.79.

#### Goldberg mental health questionnaire

The Goldberg Mental Health Questionnaire, developed by Goldberg in 1979, is a self-report questionnaire consisting of 28 questions that assess four dimensions of mental health. These dimensions include physical symptoms, anxiety and insomnia, social dysfunction, and depression, with each dimension containing seven items. Respondents rate each item on a four-point Likert scale ranging from 0 to 3. Consequently, the score for each dimension falls within the range of 0 to 21, with higher scores indicating greater disorder. On average, the questionnaire takes approximately 10 to 12 minutes to complete. Goldberg and Hiller (1979) confirmed the construct and content validity of the tool and reported the reliability of the anxiety, depression, physical symptoms, and social dysfunction dimensions using the Cronbach’s alpha method as 90%, 94%, 89%, and 80%, respectively. In Iran, the validity of the questionnaire was confirmed in Kazemi et al.‘s research using exploratory and confirmatory factor analysis for construct validity. The reliability of the questionnaire was also tested in the same study, with Cronbach’s alpha reported as 0.79 [42]. In the present study, Cronbach’s alpha for this tool was found to be 0.93.”

### Data implementation and data analysis

Necessary arrangements were made to obtain a license from the University of Rehabilitation Sciences and Social Health and the General Directorate of Education of Tehran Province. In Tehran, Students aged 14 to 18 study in high schools. There are about 600 high schools in this city and every school has a Telegram or WhatsApp group in which all students, teachers and school staff are members and important news and announcements are shared there. Porsline is an online questionnaire design software. Using an online questionnaire helps researchers save time and money while collecting a large amount of information in a short period. In this research, after designing the online questionnaire, the link was provided to school managers or other staff members in Tehran. They were requested to explain the research and share the questionnaire link on their respective social media groups. Students who expressed readiness to participate were given access codes to the questionnaire link, provided their parents had consented to their involvement in the research. Upon reaching an appropriate sample size, data collection was concluded. Data analysis was performed using SPSS software version 24, employing Pearson’s statistical tests and linear multiple regression to examine the relationships between variables. This study adhered to all ethical principles of research. Prior to participation, informed consent was obtained from all participants, where the purpose of the research, confidentiality measures, voluntary participation, and withdrawal procedures were explained. Arrangements were made with school officials and students’ parents, and students participated in the research with their parents’ consent. Furthermore, participants were assured that their responses would remain confidential.

## Result

The mean age of the students was 16.52 years with a standard deviation of 3.75. Of the participants, 61.08% were girls and 38.91% were boys. The demographic characteristics of the students are presented in Table 1.

**Table 1:** Demographic profile of participants

Variables		Frequency (%)
Gender	Girl	463 (61.08)
	Boy	295 (38.91)
Education	10th grade	169 (22.29)
	11th grade	312 (41.16)
	12th grade	277 (36.54)
Field of Study	mathematics field	209 (27.57)
	Experimental field	361 (47.62)
	Humanities	188 (24.8)
Religion	Islam	712 (93.93)
	Religions other than Islam	46 (6.06)
Socio-economic status	High	118 (15.56)
	Medium	429 (56.59)
	Low	211 (27.83)

Table 2 presents the descriptive statistics of the variables internet addiction, academic resilience, and mental health, including mean, standard deviation, skewness, and kurtosis indicators. The absolute values of skewness and kurtosis for all variables are within the recommended thresholds proposed by Kleine (not exceeding 3 and 10, respectively), indicating a normal distribution of the variables. Therefore, parametric statistical tests were utilized accordingly.

**Table 2:** Descriptive indicators of research variables

Variables	M	SD	Skewness	Kurtosis	1	2	3
Internet Addiction (1)	38.73	5.12	0.081	2.47	1		
Academic Resilience (2)	49.36	6.02	0.312	3.22	^**^0.628-	1	
Mental Health (3)	43.21	3.31	1.26	1.98	^**^0.589-	^**^0.481	1

^**^P < 0.001

In order to investigate the relationship between the variables, Pearson’s statistical test was used. Based on the information in Table 2, the results showed that Internet addiction (*r*=-0.589) has a negative and significant relationship with students’ mental health at the 0.001 level. Conversely, resilience (*r* = 0.481) exhibits a significant positive relationship with students’ mental health. Additionally, the relationship between Internet addiction and academic resilience was found to be negative and significant (*r* = -0.628).

To predict students’ mental health based on internet addiction and resilience, multiple regression analysis was conducted simultaneously. The internet addiction variable and components of academic resilience (Communication skills, future orientation, and problem-oriented/ positive orientation) were entered into the model. The summary of the results of the multiple regression analysis is reported in Table 3.

**Table 3:** The results of multiple linear regression analysis to explain the mental health of students

Model	Unstandardized Coefficients		Standardized Coefficients		Sig
	B	Std. Error	Beta	T	
Constant	10.14	3.274		3.428	0.002
Internet addiction	-0.271	0.029	-0.398	-8.323	0.001
Communication skills	0.221	0.037	0.653	9.673	0.001
future orientation	0.372	0.039	0.574	10.31	0.001
problem-oriented/ positive orientation	0.478	0.028	0.670	11.022	0.001
Model results	*R* = 0.764 R^2=^0.53 Ad.R^2^ = 0.49 F = 581.86 Sig = 0.001				

R^2^ = R-squared Ad.R^2^ = adjusted R-squared

In Table 3, the linear multiple regression coefficients of internet addiction, components of academic resilience, and students’ mental health is reported as 0.764. These variables predict a total of 53% of students’ mental health changes. In this study, G*Power 3.1.9.7 software was used to calculate actual power. Effect size = 1.04, α = 0.5, ß=α0.2, and the number of predictor variables = 2 were entered into the software, and the actual power was calculated to be 0.81.

The results of the F statistic (581.86) at the 0.001 level indicate that the variables of internet addiction and components of academic resilience (Communication skills, future orientation, and problem-oriented/ positive orientation) collectively predict the mental health of students. To investigate this, linear multiple regression analysis using the simultaneous entry method was employed. Considering that the tolerance level index for all variables was less than 1 and the variance inflation factor index was less than 3, as a result, all of them can be used in regression analysis.

The effect of internet addiction on mental health (ß=0.39) is negative and significant at the 0.001 level, and the effect of components of academic resilience (Communication skills, future orientation, and problem-oriented/ positive orientation) on mental health (ß=0.653, ß=0.574, and ß=0.670) is positive and significant at the 0.001 level. Therefore, internet addiction has a negative correlation with students’ mental health. This means that with the increase in internet addiction among students, their mental health decreases significantly. Conversely, resilience demonstrates a positive correlation with students’ mental health, suggesting that higher levels of resilience correspond to better mental health outcomes.

## Discussion

The present study was conducted with the aim of investigating the role of internet addiction and academic resilience in predicting the mental health of high school students in Tehran. The results showed that these two variables predict a total of 51% of students’ mental health changes. There is a negative and significant relationship between the variables of Internet addiction and the mental health of students, and a positive and significant relationship between the variables of academic resilience and the mental health of students.

The findings of linear multiple regression in the study indicated that internet addiction (with the presence of components of academic resilience as covariates) has a negative relationship with the mental health of students. This means that with the increase in internet addiction among students, their mental health decreases significantly. The results of this part of the research aligned with the studies of Bagheri, Ebrahimi, Khojasteh, and Veisani, which were conducted separately from each other [1, 32, 43, 44].

For instance, the results of Bagheri et al.‘s study showed that Internet addiction during the outbreak of COVID-19 damaged students’ mental health, and they were exposed to the risk of depression and anxiety [32]. Additionally, the findings of Ebrahimi and Naseri’s study indicated that social networks have a significant relationship directly with the mental health of students, and the high use of social networks will decrease their mental health [43]. The results of the study conducted by Khojesteh revealed a significant relationship between internet addiction and students’ mental health, including subscales of depression, anxiety symptoms, social functioning, sleep disorder, and physical symptoms, as well as spiritual health, including subscales of existential health and religious health in high school. Hence, it is recommended to provide regular awareness and hold specialized workshops for students, parents, and school staff to prevent mental and psychological damage from internet addiction [44]. The results of the study by Veisani et al. also confirmed that excessive use of the Internet has a significant relationship with severe depression, indicating the necessity of an educational program for students in high schools and universities regarding the correct use of the Internet [45].

On the other hand, in the present study, it was found that components of academic resilience (with the presence of internet addiction as a covariate) have a positive correlation with students’ mental health. This indicates that a higher level of resilience in students results in a higher level of mental health. The results of this part were also consistent with the research of Mesman, Ghanifar, Kehrizi, and Mortazavi, which were conducted separately. It was also determined that resilience is a vital issue in the study of psychopathology in children and adolescents, and resilience is strongly related to the mental health of children and adolescents [34, 46–48].

Mesman et al., in their systematic review study, concluded that higher levels of resilience are associated with fewer psychological problems [34]. The results of Ghanifar et al.‘s study also showed that resilience is a suggestive subscale of mental health, which has the most predictive effect on social functioning and the least predictive effect on physical symptoms [48]. In the study of Kahrizi et al., it was also determined that the variables of resilience have a direct effect on emotional health and an indirect effect on the level of satisfaction with life. The first effect of improving one’s capabilities in the field of resilience is reducing mental and emotional problems, as well as increasing the level of mental health. Consequently, these improvements lead to an increase in the level of satisfaction of a person with life [46]. Also, the results of Mortazavi et al.‘s study, which was conducted by selecting nine studies and performing meta-analysis, showed that the relationship between resilience and mental health is moderate based on Cohen’s table. This research indicates that people with high levels of resilience maintain their psychological health in stressful and unfortunate situations [47].

### Limitations

The limitations of the present study included a limited statistical population consisting solely of high school students in Tehran, and data collection was based on available sampling and limited to self-report and internet questionnaires. Therefore, caution should be exercised when generalizing the results to other age groups and regions.

Another limitation of the research was that not all variables identified in the literature review as predictors of students’ mental health, such as important stressors in students’ lives, were included in the study.

## Conclusion

The results of the present study showed that the variables of internet addiction and resilience can predict the mental health of students. Therefore, it is possible to pay attention to these factors in developing educational, preventive, and therapeutic programs for this age group. To better address these issues, prevention and intervention models should be developed by healthcare personnel, experts in education, mental health, policy makers, clinical psychologists, and other stakeholders. These programs have the potential to improve the mental health of high school students, thereby promoting psychological well-being and preventing harm during adolescence, youth, and adulthood. In cases of injury, the spread of harm can be mitigated by implementing intervention programs based on these variables.

## Data Availability

The data that support the findings of this study are available from the corresponding author upon reasonable request.
